# Interfacility Patient Transfers During COVID-19 Pandemic: Mixed-Methods Study

**DOI:** 10.5811/westjem.20929

**Published:** 2024-09-10

**Authors:** Michael B. Henry, Emily Funsten, Marisa A. Michealson, Danielle Albright, Cameron S. Crandall, David P. Sklar, Naomi George, Margaret Greenwood-Ericksen

**Affiliations:** * Creighton University Arizona Health Education Alliance, Maricopa Emergency Medicine Residency, Phoenix, Arizona; † University of New Mexico, School of Medicine, Albuquerque, New Mexico; ‡ University of New Mexico, School of Medicine, Department of Emergency Medicine, Albuquerque, New Mexico; § Arizona State University, College of Health Solutions, Phoenix, Arizona; a Equal contribution

## Abstract

**Introduction:**

The United States lacks a national interfacility patient transfer coordination system. During the coronavirus 2019 (COVID-19) pandemic, many hospitals were overwhelmed and faced difficulties transferring sick patients, leading some states and cities to form transfer centers intended to assist sending facilities. In this study we aimed to explore clinician experiences with newly implemented transfer coordination centers.

**Methods:**

This mixed-methods study used a brief national survey along with in-depth interviews. The American College of Emergency Physicians Emergency Medicine Practice Research Network (EMPRN) administered the national survey in March 2021. From September–December 2021, semi-structured qualitative interviews were conducted with administrators and rural emergency clinicians in Arizona and New Mexico, two states that started transfer centers during COVID-19.

**Results:**

Among 141 respondents (of 765, 18.4% response rate) to the national EMPRN survey, only 30% reported implementation or expansion of a transfer coordination center during COVID-19. Those with new transfer centers reported no change in difficulty of patient transfers during COVID-19 while those without had increased difficulty. The 17 qualitative interviews expanded upon this, revealing four major themes: 1) limited resources for facilitating transfers even before COVID-19; 2) increased number of and distance to transfer partners during the COVID-19 pandemic; 3) generally positive impacts of transfer centers on workflow, and 4) the potential for continued use of centers to facilitate transfers.

**Conclusion:**

Transfer centers may have offset pandemic-related transfer challenges brought on by the COVID-19 pandemic. Clinicians who frequently need to transfer patients may particularly benefit from ongoing access to such transfer coordination services.

Population Health Research Capsule What do we already know about this issue?

*Interfacility patient transfers are challenging for clinicians to arrange in rural and limited resource settings. This was exacerbated by the COVID-19 pandemic.*

What was the research question?

*Did transfer coordination centers improve the clinician experience of arranging patient transfers during the pandemic?*

What was the major finding of the study?

*Survey results and interviews suggest transfer centers may have offset pandemic transfer challenges.*

How does this improve population health?

*Clinicians who frequently transfer patients may benefit from ongoing access to transfer coordination services.*

## INTRODUCTION

In early 2020, critically ill patients with coronavirus disease 2019 (COVID-19) overwhelmed many hospitals and emergency departments (ED) across the United States. Surges were unpredictable. Patients overran some hospitals, while other facilities made significant preparations for COVID-19 patients who never arrived.^1^ Smaller hospitals needed to transfer patients to facilities with more beds, staffing, and specialist services. The unpredictable distribution of patients and bed availability placed strain on some larger regional receiving hospitals, leading some states and health systems to identify mechanisms to “load-level” patient transfers among receiving facilities.^2^

Historically, interfacility patient transfers between small or rural hospitals and larger regional hospitals has offered access to care that may not otherwise have been available. Hospitals in the US transfer over one million patients for admission annually,^3^ and nearly every US hospital participates in the transfer process either as a receiver, a sender, or both.^4^ These transfers, driven by limited bed capacity, the need for specialty services, or a lack of certain diagnostic modalities, are challenging for referring hospital clinicians.^5^

National efforts to track available hospital capacity and coordinate transfers during the early COVID-19 pandemic were seen as largely disorganized and unreliable, leading many states, cities, and hospitals to develop their own systems.^6^ The Arizona Department of Health Services created the Arizona Surge Line, a centralized system staffed by transfer coordinators with access to updated bed and ventilator capacity data around the state.^2^ New Mexico created a Central Command Center to coordinate placement of critical care patients through a “hub-and-spoke” model.^7^ Hospitals in Boston, New York City, Chicago, Washington, and Minnesota made similar efforts.^1^ ^,^ ^8^ ^–^ ^12^

While viewpoints and lay press articles have explored transfer center efficacy, research describing the reach of transfer centers and their impact, as perceived by clinicians, policymakers, and hospital leadership are lacking. We used a mixed-methods approach, including a national survey to describe access to transfer centers before and during COVID-19, and semi-structured interviews with stakeholders in two states to generate an in-depth picture of the function and the impact of transfer centers. We hypothesized that implementation of a new transfer center would lead to easier transfer processes.

## METHODS

We conducted an explanatory, mixed-methods study with a national, web-based survey to capture the transfer-center experiences in a variety of emergency care practice settings followed by semi-structured qualitative interviews with sending facility clinicians and administrators in two states (Arizona and New Mexico) to provide deeper insight into the experiences of sending facilities. Both states established new interfacility transfer coordination systems during peak COVID-19. The first author’s institutional review board approved the study design.

### Web-based National Survey

A multidisciplinary team of Arizona- and New Mexico-based researchers from a mix of urban and rural hospitals developed and revised the survey. Attending physicians with clinical, administrative, and research experience who practice in referring and receiving hospital settings pilot-tested the survey to ensure question-and-response relevance and clarity. Published guidelines informed survey development.^13^ ^,^ ^14^

We administered our survey using the American College of Emergency Physicians Emergency Medicine Practice Research Network (EMPRN), a nationwide cohort of 765 emergency physicians who have volunteered to answer short research surveys several times a year. The survey included questions on practice setting, transfer center presence during COVID-19, and transferred patients’ characteristics (Appendix A). No incentives were offered for participation. The EMPRN sent survey invitations on March 3, 2021, with three additional reminder emails over a six-week window.

#### Variables and data analysis

We calculated survey response rate based on the number of EMPRN participants who were emailed the survey (765) and the number of submitted survey responses. No surveys were excluded due to item nonresponse, since all but one of the questions were required for survey submission. We collected ordinal responses for yearly ED volume, inpatient bed capacity, and views on the future utility of transfer coordination systems and Likert-scaled responses for perception of COVID-19 impact and transfer metrics. We defined *sending* facilities as those that *always* or *mostly* tended to transfer patients out compared to receiving patients. We defined *receiving* facilities as those that *always* or *mostly* tended to receive transfers. We defined a transfer center as a “new/expanded centralized entity (such as a call center) created to coordinate interfacility transfers.” In addition to descriptive characteristics, our primary outcome measure of interest was the transfer center effect on patient transfers during COVID-19. We compared sending and receiving respondents, facility characteristics, effects of the COVID-19 pandemic, and perceptions of transfer centers using chi-square, Student *t*-test, Spearman’s rho, Wilcoxon signed-rank tests, and Mann-Whitney tests, as appropriate. We used a two-tailed type I error rate of 5% to determine statistical significance. We used JASP 0.14.1.0 (University of Amsterdam, Netherlands) for statistical analyses.

### Qualitative Interviews

We conducted semi-structured interviews to further explore clinician and policymaker perceptions of interfacility transfers before and during the pandemic, along with the benefits and challenges of the new transfer systems. Researchers iteratively developed the interview guides with input from physicians frequently involved in the transfer process. Clinicians who gave input on the development of the interview tool were not included among the final interviews. The interview questions focused on the organization of and overall challenges associated with interfacility transfers and on the perceived impacts of transfer centers on the transfer processes (Appendix B).

Three authors conducted the interviews between September–December 2021. Clinicians who gave input on the survey tool helped identify the first interviewees. We then used snowball sampling to recruit clinicians from rural (sending) hospitals that frequently needed to transfer patients to other facilities and administrators tasked with implementing and running the transfer centers during COVID-19. We interviewed five clinicians and three administrators from New Mexico and six clinicians and three administrators from Arizona before thematic saturation was reached and no further participants were recruited. Interviews generally ranged from 30–60 minutes in length. With permission, we recorded the interviews, saved them securely, and transcribed them using Rev transcription services (Rev.Com Inc, San Francisco, CA). We assigned alphanumeric identifiers to transcripts for confidentiality. Each of the three interview-team members independently coded the same administrator and clinician interview to ensure concordance and to develop a coding structure before inductively coding and analyzing themes on the remaining interviews. We used an iterative process throughout the analysis to ensure reliability in thematic category development.

## RESULTS

### Survey

**Figure 1.**
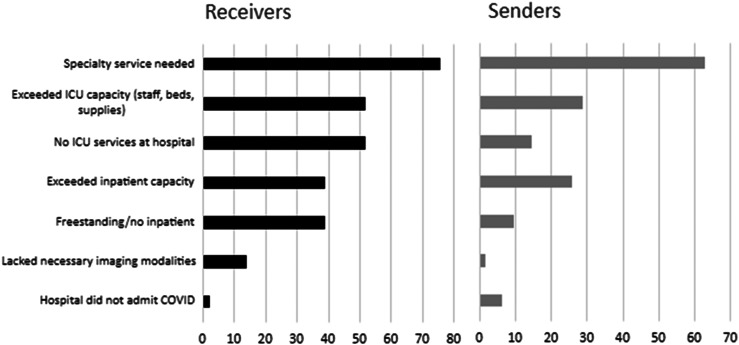
Perceived reasons necessitating patient transfers during the COVID-19 pandemic, receiving vs sending facilities (% of respondents). *COVID*, coronavirus 2019; *ICU*, intensive care unit.

A total of 141 physicians (of 765 who were sent the survey, 18.4% response rate) from 39 states responded to the EMPRN survey. Respondent average age was 53, and most were White and male. Facilities that primarily transferred patients to larger centers (ie, sending facilities) tended to have lower yearly ED volumes (*P* < 0.001) and less inpatient bed capacity (*P* < 0.001) compared to facilities that primarily received patients in transfer (ie, receiving facilities) (Table 1). Lack of specialty services was the most common reason for transfer reported among both senders and receivers; other reasons included inadequate local inpatient- and intensive care unit (ICU) capacity (Figure 1).

**Table 1.** Demographics and general characteristics of respondents and respondent facilities from national EMPRN^*^ survey.

		Overall	Receivers	Senders	*P*-value
Total		141	79	62	
Unique states		39	35	25	
Mean age (years)		53.4	52.1	55.0	0.11
Ethnicity (%white)		82.5	79.2	86.7	0.26
Gender (%male)		80.9	79.7	82.3	0.71
Yearly volume (%)	<10k patients	5	0	11.3	<0.001
	10–30k	20	11.5	30.6	
	30–60k	35.7	34.6	37.1	
	>60k	39.3	53.8	21	
Inpatient beds (%)	None (freestanding)	3.5	1.3	6.5	<0.001
	<25 beds	7.8	0	17.7	
	25–99	9.9	2.5	19.4	
	100–299	34.8	26.6	45.2	
	300–500	22.7	32.9	9.7	
	>500	21.3	36.7	1.6	
COVID-19 impact, Likert 1–5 (SDEV)		3.90 (0.905)	4.04 (0.884)	3.73 (0.908)	0.05
Pre-COVID-19 transfer	City/county/region	10.1	11.7	8.1	0.22
center (%)	State	4.3	3.9	4.8	
	Hospital-based	15.8	18.2	12.9	
	No	65.5	59.7	72.6	
	Don’t know	4.3	6.5	1.6	
New or expanded	City/county/region	5.7	6.3	4.8	0.21
transfer center (%)	State	9.2	13.9	3.2	
	Hospital-based	11.3	8.9	14.5	
	No/unchanged	59.6	51.9	69.4	
	Don’t know	11.3	15.2	6.5	

* *EMPRN*, Emergency Medicine Practice Research Network.

Most respondents reported moderate-to-high perceived severity of COVID-19 impact in their areas (mean 3.9 with 1 being the lowest severity and 5 the highest severity). Physicians at receiving facilities perceived the severity of the COVID-19 pandemic to be greater than sending physicians (*P* = 0.05), see Table 1. Most respondents also reported transfers over greater distances (mean 3.58) and increased number of transfer partners (mean 3.54) required to accomplish patient transfers during the COVID-19 pandemic compared to prior (mean 3.00, indicating no change). Reported COVID-19 intensity appeared to be correlated to distance of transfers (Spearman’s rho 0.141, *P* = 0.10) and number of transfer partners (Spearman’s rho 0.140, *P* = 0.10) although not statistically significant.

Most respondents reported no access to a transfer center prior to the COVID-19 pandemic, or implementation of a new or expanded transfer center in response to COVID-19 (Table 1). A total of 37 respondents (30%) reported implementation or expansion of a transfer coordination center in response to the COVID-19 pandemic. Those who had a new or expanded center reported similar amounts of overall effort (mean 2.91, where a mean = 3 indicates no change, Wilcoxon rank-sum test, *P* = 0.55) and time to achieve transfers (mean 3.17, Wilcoxon *P* = 0.32). In comparison, those who did not have a new or expanded center generally reported a higher difficulty (mean 3.89, Wilcoxon rank-sum test *P* < 0.001) of transferring patients because of the COVID-19 pandemic.

Of those with access to new or expanded transfer centers during COVID-19, most saw utility in having these transfer services available in the future. Among 36 respondents with new or expanded transfer centers, 15 (42%) thought they would be useful in the future but only in emergency circumstances and 16 (44%) thought they would be useful in all situations. In comparison, of 83 respondents without transfer centers, only 17 (20%) thought transfer centers could help in future emergencies, 35 (42%) thought they would be useful in all situations, and 31 (37%) thought these would not be helpful in the future.

### Interviews

Interviews with administrators and sending facility clinicians provided additional insight on the utility of transfer centers. The 17 interviewees included five clinicians and three administrators from New Mexico and six clinicians and three administrators from Arizona. The interviewees were mixed in gender and age. The 11 clinicians included six working on tribal health sites of varying sizes (a large number of rural facilities in the region serve Native American tribes), one at a non-tribal nonprofit critical access hospital, two at mid-sized community nonprofit hospitals, one at several freestanding EDs, and one at a rural community teaching hospital.

We identified four primary themes related to the functioning and utility of transfer centers, including the following: 1) limited resources for facilitating transfers even before COVID-19; 2) increased number of and distance to transfer partners during the COVID-19 pandemic; 3) generally positive impacts of transfer centers on workflow; and 4) the potential for continued use of centers to facilitate transfers.

#### Theme 1: Limited resources to coordinate transfers

Interviewees reported that, prior to and during the COVID-19 pandemic, transfer coordination placed a large burden on clinicians and staff at sending facilities. One respondent noted that they spent *“half my shift on the phone,”* and another called *“as many as 10 to 12 facilities”* before completing a transfer. Without centralized access to learn about which receiving facilities had needed beds and specialty services, *“you had no way of knowing that until you actually talk to somebody.”*

Respondents at sending facilities reported a variety of reasons for patient transfer. A respondent summarized the relationship between resources and decisions to transfer:

There are three [reasons], one is lack of capacity… The other reason is called ‘service is not available’… And the third reason is higher level of care… that the patient is too sick for us and our system.

Before transfer center implementation, decisions were often based on sending clinicians’ personal relationships and knowledge of receiving facilities, to the extent that one interviewee noted their *“first-name relationships”* with those at receiving facilities.

#### Theme 2: Increased transfer complexity during COVID-19

Survey respondents reported increased numbers of transfer partners and increased distances of transfers, suggesting increasing complexity of patient transfer as an early result of the pandemic. Interview respondents elaborated on the complexities of this expanded transfer area, with one noting that *“transferring patients much farther distances”* places strain on *“the patient and onto the family and their support network.”* Another interviewee had a similar number of transfers as pre-pandemic but *“the complexity increased, the number of phone calls increased.”* Many needed to identify new receiving facilities beyond their normal transfer partners to access the needed level of care, specifically ICU beds. A respondent reported,

Before this [COVID-19 pandemic], I’ve never had to transfer for bed availability, but we did start having to do that for ICU beds. [For these reasons] “length of stay in the emergency department has gone up dramatically.

#### Theme 3: The positive impact of transfer centers

Almost all respondents noted that the transfer centers decreased the burden of patient transfer, providing an important resource in maintaining workflow. A primary impact was reduction in time spent on the phone, with one respondent liking that they *“only had to dial one number, give some basic info…you only had to have one conversation with one doctor… it dramatically decreased administrative work.”* Another interviewee noted, *“better flow throughout our emergency department so that other patients could be seen and cared for as well.”* Even those who did not see a big impact on their transfer process acknowledged the possibility that the centers helped subtly:

As the transferring physician, I did not see much change… Now, what I can’t tell you is what it would be like, given the ongoing surges, if there had been an absence of that call center, things could have been much worse… Maybe no change was a good thing, because the alternative was that things would’ve declined in a very bad way.

#### Theme 4: Transfer centers as a potential policy solution

The transfer center experience led interviewees to consider how this arrangement may contribute to improved care delivery beyond the pandemic. Despite lower numbers of COVID-19 patients at the time of interview, one clinician noted transfers still *“taking quite a bit of time…transfer center assistance could still be very helpful… even though technically the COVID crisis has subsided.”*

One respondent suggested a standing statewide transfer center could assist sending facilities and increase patient agency: *“One phone number that you call for any transfers within your state… you can put that patients’ preference is transferred to X hospital or to stay near home.”* Others stated they would like to *“expand this out to other disease states other than just COVID.”* Another wanted to see *“a [phone] line at a minimum that dealt with all ICU beds within a region…able to know what hospitals had what services and put you in touch with them rather than… [taking] away from patient care.”*

Meeting the needs of both facilities and communities requires significant planning and coordination. One respondent noted,

Outside of pandemic times there is not a well-coordinated transfer architecture for getting patients from deeply rural to urban centers and tertiary care centers… [I am] hopeful that we can pull together a well-coordinated transfer framework.

Another commented,

When we’re seeing higher volumes and pressed for time and resources in the ER, anything that could be done to facilitate patients ultimately getting the care they need is a huge benefit…if we can optimize the resources within the state to allow for coordination across the hospital systems…that would be ideal.

A subset of respondents was less enthusiastic about transfer centers outside the pandemic context. One stated, *“I don’t feel like I need it during non-crisis times, personally. I usually have been able to transfer patients pretty quick.”* Others noted interrelated challenges that would need to be overcome before fully realizing the benefits of a transfer center: *“We’re so rural…we also still have to arrange for flight and for the actual transportation.”* Additional and expanded respondent quotes are provided in Table 2.

**Table 2.** Additional qualitative findings and extended-length quotes.

Theme	Quote
Limited resources to coordinate transfers	You make all your own phone calls. So I’ve spent up to half my shift on the phone, randomly dialing phone numbers for non-COVID patients before.
	There are times when they call as many as 10 to 12 facilities before you can find an acceptable bed…so the more time spent trying to find an appropriate facility for patient, the fewer other patients that that provider can see.
	I’ve spent more time on the phone … trying to get people transferred due to COVID than I ever thought was possible.
	Even if a place said that they have specialty services covering specialty X, they may not have it that day or that week and you had no way of knowing that until you actually talk to somebody. You wouldn’t know who had the type of bed available that you were looking for. So you’d [make] multiple phone calls say requesting an ICU bed until you found somebody who had an open ICU bed. And so just a lot of redundancy of work that took away from patient care time.
	There are three [reasons], one is lack of capacity, because we can’t admit that many patients because we don’t have enough nurses. The other reason is called “service is not available”, which means we just don’t have the service, we don’t have the specialist, we don’t have the MRI, we don’t have the CT, our CTs went down. There’s a lot of things we don’t have. We don’t have platelets, we don’t have dialysis, don’t have anyone to do a cardiac check. Either we don’t have the specialist, we don’t have the equipment, but we don’t have the service that service is not available. And the third reason is higher level of care. And that’s that the patient is too sick for us and our system, which is different than service is not available. Service is not available would be if we had a neurologist and MRI, we could probably keep the patient. So those are only three reasons we send people out.
	We have first name relationships with some of the attending physicians, and they’re more familiar with our setting and our limitations
Increased transfer complexity during COVID	We are now transferring patients much farther distances, which is very significant onto the patient and onto the family and their support network. Many patients are being transferred now to locations where they don’t have a ride home
	I think the proportion of transfers was probably similar. But, the complexity increased, the number of phone calls increased
	We don’t have a lot of specialists and so it is not infrequent that we have to transfer for certain specialists but before this [COVID pandemic], I’ve never had to transfer for bed availability, but we did start having to do that for ICU beds
	Our length of stay in the emergency department has gone up dramatically, compared from even in the last month, we were up 109 minutes on average for patients that were being admitted
The positive impact of transfer centers	I think where it was most notable was just the fact that you or a secretary only had to dial one number, give some basic info. And then once there was an accepting hospital, then as a physician, you only had to have one conversation with one doctor. Sometimes not even that. And so from sort of that end of the administrative work, it dramatically decreased administrative work.
	So patients having to wait less time to get to an appropriate inpatient facility would be beneficial and then would also allow us to have better flow throughout our emergency department so that other patients could be seen and cared for as well
	From my end, as the transferring physician, I did not see much change… Now, what I can’t tell you is what it would be like, given the ongoing surges, if there had been an absence of that call center, things could have been much worse. So in the back of my mind, I think about that. Maybe no change was a good thing, because the alternative was that things would’ve declined in a very bad way
Transfer centers as a potential policy solution	We’re still finding that the process to find an appropriate facility is taking quite a bit of time. And so that transfer center assistance could still be very helpful even now because of the fact that the state is just seeing limited bed availability, even though technically the COVID crisis has subsided.
	One phone number that you call for any transfers within your state. And that you can put that patients’ preference is transferred to X hospital or to stay near home or whatever [their preference] is.
Transfer centers as a potential policy solution	I think that I would love to expand this out to other disease states other than just COVID. I think having that centralized communication system seemed to be a very effective manner.
	Having a line at a minimum that dealt with say all ICU beds within a region. But ideally one line that was able to know what hospitals had what services and put you in touch with them rather than having hospital staff spending most of their time dialing numbers and away from patient care
	I would be fully in favor of a robust, well-supported transfer mechanism. My impression is that outside of pandemic times there is not a well-coordinated transfer architecture for getting patients from deeply rural to urban centers and tertiary care centers… very hopeful that we can pull together a well-coordinated transfer framework, which revolves around transfer centers
	Even if they cannot cover every single transfer, especially, when we’re seeing higher volumes and pressed for time and resources in the ER, anything that could be done to facilitate patients ultimately getting the care they need is a huge benefit. I think there’s also sort of a sense that if we can optimize the resources within the state to allow for coordination across the hospital systems, just here for these patients, that would be ideal as well
	I don’t feel like I need it during non-crisis times, personally. I usually have been able to transfer patients pretty quick
	We’re so rural. There’s nowhere… When it takes us so long to get a bed, we also still have to arrange for flight and for the actual transportation. So, if it takes 22 hours to get a bed, and then I don’t have a flight team available for six hours, that’s 28 hours before the patient leaves my emergency department, you know?

*CT*, computed tomography; *COVID*, coronavirus 2019; *ICU*, intensive care unit; *MRI*, magnetic resonance imaging.

## DISCUSSION

In this mixed-methods study, we hypothesized that physicians participating in patient transfers during COVID-19 would find utility in having access to transfer coordination centers. In our national survey, those without access to transfer centers reported a significant increase in the difficulty of executing transfers during COVID-19 surges, contrasting to respondents with access to new transfer coordination services reporting no significant change in effort or time to organize a patient transfer. This suggests that transfer center implementation may have offset pandemic-related transfer challenges. Respondents who had experience with transfer centers also were more supportive of their ongoing use, particularly in future health crises.

Our qualitative interviews in Arizona and New Mexico, two states where transfer centers were implemented, shed additional light on their benefits while identifying areas for future improvement. With few exceptions, these clinicians found the transfer center services to be helpful, specifically in reducing transfer workflow complexity. Since the completion of this study, feedback in Arizona was so positive that the state evolved its temporary transfer center into the federal grant-funded “AZ REACH” system, administered by the Arizona Poison and Drug Information System.^15^

Most US healthcare practitioners do not work in areas served by transfer coordination centers, consistent with our survey results.^1^ ^,^ ^2^ ^,^ ^6^ ^–^ ^12^ In the wake of the September 11, 2001, terrorist attacks, attempts were made to create a national centralized system but were never widely implemented.^6^ While some regions have transfer patterns for specific conditions such as trauma and acute myocardial infarction,^16^ ^,^ ^17^ transfers generally occur through informal, loosely coordinated regional networks that suffer from fragmentation and poor communication.^4^ ^,^ ^5^ Our study concurs with prior research finding that the laborious transfer process requires the sending clinician to identify an accepting facility using their knowledge of historical transfer patterns and informal relationships, and the complex coordination and multiple phone calls can distract from patient care.^5^ It is possible that existing transfer services were available to some of our respondents prior to COVID-19 but clinicians only became aware of them due to the strains of the pandemic.

Our survey found receiving centers reported a greater perceived impact of COVID-19. Receiving hospitals are generally located in urban areas, which experienced the first waves of COVID-19 during a time of intense uncertainty and fear. This, along with subsequent surges of rural transfers to urban facilities, may have influenced the perspectives of receiving hospital respondents.^18^ It is also possible that sending facilities and rural respondents from the survey may have lived in regions less impacted by COVID-19, or that closer relationships between rural facilities and their local communities may yield increased resilience in responding to crises.^19^ However, the qualitative interviews revealed that significant stresses from COVID-19, including with transfers, also affect clinicians at smaller facilities. This is likely exacerbated by the struggles of rural facilities to hire physicians who often choose to work in urban centers,^2^ while inability to retain staff and rising rural hospital closures^21^ place an increased burden among those who remain. Lack of specialist services and inpatient/ICU capacity were the top reasons prompting transfers per sending and receiving physicians, similar to what was reported in prior studies.^5^

Small and rural hospitals have had longstanding challenges transferring patients with time-sensitive conditions, which were amplified during COVID-19. Transfer centers seem to hold great promise in making patient transfer less onerous. As the US healthcare system continues to struggle with worsening staffing issues and patient crowding, transfer centers may be one part of the larger solution to get patients needed care.

## LIMITATIONS

The low response rate in the national survey potentially limits interpretation and introduces bias. The strict requirement to complete nearly all questions on the survey may also have led to unit non-response among some physicians. While we tried to improve response rates with multiple email reminders and a shorter survey, other proven strategies could have been considered, such as reaching out via postal mail or including incentives.^22^ ^,^ ^23^ It does appear that survey response rates may be decreasing over time and may also vary by medical specialty, with some having low response rates below 30%.^24^ ^,^ ^25^

Since EMPRN requests surveys be as short and simple as possible to encourage participation, we further explored clinicians’ experiences via key informant interviews. The small pool of interview respondents limited our qualitative data, while the use of snowball sampling from two neighboring, largely rural states (and many tribal sites) may have limited the diversity of viewpoints and external generalizability. Future studies could incorporate expanded interviews to gather perspectives from clinicians in different states and practice settings.

## CONCLUSION

The widespread strain on the US healthcare system during the COVID-19 pandemic manifested significant challenges in interfacility patient transfers. Clinicians at small, rural facilities in Arizona and New Mexico found centralized transfer coordination centers to be helpful. In the future, other states could consider trialing implementation of similar services, both in crisis and non-crisis times.

## Supplementary Information



