# Supplementary material for: Interfacility Patient Transfers During COVID-19 Pandemic: Mixed-Methods Study
Source: West J Emerg Med. 2024 Sep 10;25(5):758–66. doi: 10.5811/westjem.20929 (PMC11418862; doi:10.5811/westjem.20929)
Supplement: Supplementary file 1 [file wjem-25-758-s001.docx]

APPENDIX A: EMPRN NATIONAL SURVEY

**Interfacility transfers during COVID-19**

Please answer the following questions about the facility where you work most of your shifts

*All questions other than #2 are required

1- How would you best estimate the usual pre-COVID ED volume?

- <10,000 visits per year
- 10-30,000 visits per year
- 30-60,000 visits per year
- >60,000 visits per year

2- What is the 5-digit ZIP code of your facility? Fill in________________.

The purpose of this is further classification as rural/urban

3- How would you best describe the INPATIENT CAPACITY at your facility?

- Freestanding (no inpatient beds)
- <25 beds
- 25-99
- 100-299
- 300-500
- Greater than 500

4- How significantly has COVID affected your facility and its surrounding region?

Not at all

- 1
- 2
- 3
- 4
- 5

Very severely

5- Prior to COVID, did you have a centralized entity to help coordinate interfacility transfer outside of your own health system?

- I don't know
- No, only informal processes to coordinate transfers
- Yes, run at the city level
- Yes, run at the county level
- Yes, run at the state level
- Other: _______________

6- For adult transfers, would you estimate that your ED/hospital:

- Almost always RECEIVES transfers >jump to question 7
- Mostly RECEIVES but sometimes sends transfers >jump to question 7
- Mostly SENDS but sometimes receives transfers >jump to question 11
- Almost always SENDS transfers >jump to question 11

**RECEIVERS**

7- During regional surges of COVID, what were the most frequent reasons sending hospitals transferred patients to your facility? Select all that apply:

- Freestanding ED, or no inpatient service
- Hospital had exceeded inpatient bed capacity
- Hospital did not offer ICU services
- Hospital had exceeded ICU capacity (staffing, beds, ventilators, dialysis, etc)
- Hospital lacked requisite imaging modalities
- Need for specialty service or care (stroke, cardiac, trauma, etc)
- N/A: our region has not experienced high incidence of COVID
- Other: _______________

8- How did COVID affect the CATCHMENT AREA from which your ED received transfers?

3= no change

MUCH SMALLER - transfers came from nearby compared to usual

- 1
- 2
- 3
- 4
- 5

MUCH LARGER - transfers came from further away than usual

9- How did COVID affect the NUMBER OF DIFFERENT facilities sending patients to your ED? 3= no change

MUCH FEWER - less different facilities than usual transferred patients to my facility

- 1
- 2
- 3
- 4
- 5

MANY MORE - more different facilities sent transfers to us than usual

10- During regional surges of COVID, were there any NEW/EXPANDED centralized entities (such as a call center) created to coordinate interfacility transfers?

- I don't know >jump to question 21
- My facility's region was not significantly affected by COVID >jump to question 21
- No, only the usual process for transfer coordination >jump to quest 15
- One was planned but never enacted >jump to question 15
- Yes, run by the hospital system >jump to question 17
- Yes, run at the city level >jump to question 17
- Yes, run at the county level >jump to question 17
- Yes, run at the state level >jump to question 17
- Other:_______________ >jump to question 21

**SENDERS**

11- During regional surges of COVID, what were the most frequent reasons your facility needed to transfer patients out? Select all that apply:

- Freestanding ED, or no inpatient service
- Hospital had exceeded inpatient bed capacity
- Hospital did not offer ICU services
- Hospital had exceeded ICU capacity (staffing, beds, ventilators, dialysis, etc)
- Hospital lacked requisite imaging modalities
- Need for specialty service or care (stroke, cardiac, trauma, etc)
- N/A: our region has not experienced high incidence of COVID
- Other: ______________

12- How did COVID affect the DISTANCE you typically had to transfer patients?

3= no change

MUCH CLOSER - could transfer patients to facilities closer than compared to usual

- 1
- 2
- 3
- 4
- 5

MUCH FURTHER - had to transfer patients much further away than usual

13- How did COVID affect the NUMBER OF DIFFERENT EDs you sent patients to?

3= no change

MUCH FEWER – fewer transfer partners than usual were needed to accept most of our transfers

- 1
- 2
- 3
- 4
- 5

MANY MORE - more transfer partners than usual were needed to accept most of our transfers

14- During regional surges of COVID, were there any NEW/EXPANDED centralized entities (such as a call center) created to coordinate interfacility transfers?

- I don't know >jump to question 21
- My facility's region was not significantly affected by COVID >jump to question 21
- No, only the usual process for identifying a facility to accept a transfer >jump to quest 16
- One was planned but never enacted >jump to question 16
- Yes, run by the hospital system >jump to question 19
- Yes, run at the city level >jump to question 19
- Yes, run at the county level >jump to question 19
- Yes, run at the state level >jump to question 19
- Other: >jump to question 21

**RECEIVERS WITHOUT CALL CENTER:**

15- During regional COVID surges, the effort required to coordinate transfers became:

3= no significant change due to COVID

Much easier/less time consuming

- 1
- 2
- 3
- 4
- 5

Much harder/more time consuming

>jump to question 21

**SENDERS WITHOUT CALL CENTER:**

16- During regional COVID surges, the effort required to transfer patients became:

3= no significant change due to COVID

Much easier/less time consuming

- 1
- 2
- 3
- 4
- 5

Much harder/more time consuming

>jump to question 21

**RECEIVERS WITH CALL CENTER**

17- How did the centralized transfer center impact the effort required to coordinate transfers:

3= transfer call center did not change difficulty of transfer coordination

Much easier

- 1
- 2
- 3
- 4
- 5

Much harder

18- How did the centralized transfer center impact length of time to transfer a patient to your facility?

3= transfer call center did not change time to transfer

Much less time

- 1
- 2
- 3
- 4
- 5

Much more time

>jump to question 21

**SENDERS WITH CALL CENTER**

19- How did the centralized transfer center impact the effort required to transfer patients:

3= transfer call center did not change difficulty of transfer coordination

Much easier

- 1
- 2
- 3
- 4
- 5

Much harder

20- How did the centralized transfer center impact length of time to transfer a patient from your facility?

3= transfer call center did not change time to transfer arrival

Much less time

- 1
- 2
- 3
- 4
- 5

Much more time

>jump to question 21

**FINAL QUESTION**

21- Do you think a state or regional centralized entity assisting coordination of interfacility transfers would be useful in the future?

- Yes, mostly during emergencies such as national disasters and pandemics
- Yes, even in non-emergency circumstances
- No, this entity did not help the transfer process (or I do not believe it would have, if we had one)
- Other comments: __________________

APPENDIX B: SEMI-STRUCTURED QUALITATIVE INTERVIEW

Provider interview tool

*Introduction:*

*My name is [name] I am a [role] at the [institution]. I am part of a research team that is hoping to learn more about the utilization of central call centers in facilitating the transfers of patients from rural to urban hospitals during the COVID pandemic. I am going to ask you some questions to better understand your experience in the transfer of patients between hospitals with and without the use of the transfer call centers. This interview* i*s voluntary and anonymous. We will record this conversation to ensure we capture all your responses, but this information will be kept confidential inside the research team.*

*There are protections in place to assure privacy, and there is minimal risk of a privacy breech. No personally identifiable information will be reported. For more information, please contact [first author] at [anonymized].*

*You will also be sent a short survey we would appreciate if you filled out immediately afterwards.*

*Do you have any questions before we begin?*

**Section I: Icebreaker**

*First, I would like to learn a little more about your experience and where you work.*

1) Please describe your job and responsibilities.

- PROBE:
- What roles do you fill in your hospital? Clinical vs admin etc
- What patient populations do you work with?

**Section II: Transfer Process**

*These questions will focus on the patient transfer process at your hospital.*

2) How did patient transfers work at your hospital in pre-COVID times?

- PROBE:
- What were common reasons for transfer?
- What factors helped facilitate, and what were barriers to transfer?
- What was your role as a physician in transfers?
- How frequently/why did you encounter difficulty arranging a transfer?
- How would you characterize the transfer process in terms of ease/difficulty and its effect on patient outcomes?
- Can you describe a typical experience of arranging a transfer prior to COVID?
- (sub probes) What worked well?
- What did not work?

3) How did patient transfers at your hospital change due to COVID?

- PROBE
- Reasons for transfer (#beds, ICU capacity, specialists)
- Frequency of transfer
- Rejected transfers requests and why
- Number of calls/amount of effort to arrange a single transfer
- Transfer delays and reasons
- Overall clinical care experience of patients needing transfer

**Section III: Interactions with transfer coordination center/surge line**

*The next few questions are going to focus on interactions with the transfer coordination center and/or surge line.*

4) How did transfer call centers impact the transfer process?

- PROBE
- How were the logistics of using the transfer center different than how transfers are normally coordinated? (for example, who interacts with the transfer center?)
- Did transfers become easier or harder? How?
- What aspects of working with the transfer centers did you like/dislike?
- How was transfer time affected by use of the transfer center?
- How was the patient experience otherwise affected by use of the transfer center?
- Patient satisfaction, outcomes, family satisfaction, timely care
- How did the presence of the transfer center affect your workflow?
- How about the effort it took for you to coordinate a transfer?
- Can you describe a typical experience of coordinating a transfer using the transfer coordination center?

5) How familiar are you with transfer scenarios in other states or regions? [if interviewees are unfamiliar with situation in other places, can skip question]

- How did your experience with your state’s transfer center during COVID surges compare to your knowledge of transfer practices in other states or areas?
- What parts of working with your state’s transfer center were helpful or unhelpful compared to what other states had access to?

**Section IV: Closing**

*Great! I have a few closing questions to round out this discussion.*

6) How has the transfer process changed since the last surge subsided?

- How would you feel about having access to a transfer coordination system in future crises?
- How about during non-crisis times?

7) If you were given the authority to make a transfer coordination system, what would it look like?

8) What didn’t we ask that you think is important regarding hospital transfers and/or the Transfer Coordination Call Centers? Is there anyone else we should talk to?

Administrator interview tool

*Introduction:*

*My name is [name] I am a [role] at the [institution]. I am part of a research team that is hoping to learn more about the utilization of central call centers in facilitating the transfers of patients from rural to urban hospitals during the COVID pandemic. I am going to ask you some questions to better understand your experience in the transfer of patients between hospitals with and without the use of the transfer call centers. This interview* i*s voluntary and anonymous. We will record this conversation to ensure we capture all your responses, but this information will be kept confidential inside the research team.*

*There are protections in place to assure privacy, and there is a minimal risk of privacy breech. No personally identifiable information will be reported. For more information, please contact [first author] at [anonymized].*

*You will also be sent a short survey we would appreciate if you filled out immediately afterwards.*

*Do you have any questions before we begin?*

**Section I: Icebreaker**

*First, I would like to learn a little more about your background, experience, and where you work.*

1) Describe your job and responsibilities prior to your involvement with COVID transfer centers

- PROBE:
- What organization did you work for?
- What roles did you fill in your position?
- Did you or your organization play any role in patient transfers?

**Section II: Transfer Centers**

*The next few questions focus on the structure of the transfer center and how that has evolved over the last year.*

2) How did your state transfer center (surge line) function during COVID-19?

- PROBES:
- How were patient transfers coordinated prior to COVID?
- How is the newly implemented system different from this?
- Can you describe a typical experience of coordinating a transfer using the transfer coordination center?
- Who was involved, how did physicians participate in the communication, etc
- What was the structure of the transfer center? (who staffed it, what organization housed it, etc)

3) How do you think the transfer centers impacted the transfer process?

- What factors helped the transfer center work well? What were barriers the transfer center faced in accomplishing its tasks?
- Ask about barriers dealing with both the receiving and sending hospitals and also the functionality of the center
- Were there any regional differences in how the system worked, in terms of barriers and facilitators?

4) Can you speak to how the transfer center changed from the start of the pandemic until now?

- PROBES:
- How has the organizational structure changed?
- How has the role of the center in coordinating transfer changed?
- What was the impetus for these changes?
- Were there times during the pandemic when the center was not active?
- What indications were there for stopping the transfer center, and then re-starting it?

5) How familiar are you with transfer scenarios in other states or regions? [if interviewees are unfamiliar with situation in other places, can skip question]

- How did your experience with your state’s transfer center during COVID surges compare to your knowledge of transfer practices in other states or areas?
- What parts of working with your state’s transfer center were helpful or unhelpful compared to what other states had access to?

**Section III: Closing**

*Great, I have few closing questions to round out this discussion.*

6) How has the transfer process changed since the last surge subsided?

- How would you feel about having access to a transfer coordination system in future crises?
- How about during non-crisis times?

7) If you were given the authority to make a transfer coordination system, what would it look like?

8) What didn’t we ask that you think is important regarding hospital transfers and/or the Transfer Coordination Call Centers? Is there anyone else we should talk to?
